# Clinical relevance of the radiation dose bath in lower grade glioma, a cross-sectional pilot study on neurocognitive and radiological outcome

**DOI:** 10.1016/j.ctro.2022.02.001

**Published:** 2022-02-08

**Authors:** Hiska L. van der Weide, Justyna Kłos, Johannes A. Langendijk, Charlotte L. Brouwer, Peter F. Sinnige, Ronald J.H. Borra, Rudi A.J.O. Dierckx, Rients B. Huitema, Sandra E. Rakers, Anne M. Buunk, Jacoba M. Spikman, Ingeborg B. Bosma, Roelien H. Enting, Merethe Blandhol, Roland K. Chiu, Anouk van der Hoorn, Miranda C.A. Kramer

**Affiliations:** a University of Groningen, University Medical Center Groningen, Department of Radiation Oncology, Hanzeplein 1, 9713GZ Groningen, The Netherlands; b University of Groningen, University Medical Center Groningen, Department of Nuclear Medicine and Molecular Imaging, Hanzeplein 1, 9713GZ Groningen, The Netherlands; c University of Groningen, University Medical Center Groningen, Department of Radiology Hanzeplein 1, 9713GZ Groningen, The Netherlands; d University of Groningen, University Medical Center Groningen, Department of Neurology, Hanzeplein 1, 9713GZ Groningen, The Netherlands; e University College Groningen, University of Groningen, Hoendiepskade 24, 9718BG Groningen, The Netherlands

**Keywords:** RT, Radiotherapy, LGG, Lower Grade Glioma, RIBD, Radiation-Induced Brain Damage, NCF, Neurocognitive Function, MRI, Magnetic Resonance Imaging, CMB, Cerebral Microbleed, CTV, Clinical Target Volume, UMCG, University Medical Center Groningen, IDH, Isocitrate DeHydrogenase, WHO, World Health Organisation, FLAIR, Fluid-Attenuated Inversion Recovery, T, Tesla, TE, Echo Time, TR, Repetition Time, TI, Inversion Time, FoV, Field-of-View, SWI, Susceptibility-Weighted Imaging, SDMT, Symbol Digit Modalities Test, TMTA, Trail Making Test part A, TMTB, Trail Making Test part B, TMTBA, Trail Making Test part B divided by A, COWAT, Controlled Oral Word Association Test, RAVLT-IR, Rey’s Auditory Verbal Learning Test – immediate recall, RAVLT-DR, Rey’s Auditory Verbal Learning Test – delayed recall, CFT, Rey Complex Figure Test, NTCP, Normal Tissue Complication Prediction, Radiotherapy, Glioma, Cerebral microbleeds, Volumetry, Neurocognitive function, Verbal memory

## Abstract

**Aim:**

To investigate the clinical relevance of the radiotherapy (RT) dose bath in patients treated for lower grade glioma (LGG).

**Methods:**

Patients (n = 17) treated with RT for LGG were assessed with neurocognitive function (NCF) tests and structural Magnetic Resonance Imaging (MRI) and categorized in subgroups based on tumour lateralisation. RT dose, volumetric results and cerebral microbleed (CMB) number were extracted for contralateral cerebrum, contralateral hippocampus, and cerebellum. The RT clinical target volume (CTV) was included in the analysis as a surrogate for focal tumour and other treatment effects. The relationships between RT dose, CTV, NCF and radiological outcome were analysed per subgroup.

**Results:**

The subgroup with left-sided tumours (n = 10) performed significantly lower on verbal tests. The RT dose to the right cerebrum, as well as CTV, were related to poorer performance on tests for processing speed, attention, and visuospatial abilities, and more CMB.

In the subgroup with right-sided tumours (n = 7), RT dose in the left cerebrum was related to lower verbal memory performance, (immediate and delayed recall, r = −0.821, p = 0.023 and r = −0.937, p = 0.002, respectively), and RT dose to the left hippocampus was related to hippocampal volume (r = −0.857, p = 0.014), without correlation between CTV and NCF.

**Conclusion:**

By using a novel approach, we were able to investigate the clinical relevance of the RT dose bath in patients with LGG more specifically. We used combined MRI-derived and NCF outcome measures to assess radiation-induced brain damage, and observed potential RT effects on the left-sided brain resulting in lower verbal memory performance and hippocampus volume.

## Highlights

• Radiation-induced brain damage as a consequence of the RT dose bath was investigated.
• Multiple MRI-derived metrics and neurocognitive function domains were analysed.
• Our novel approach accounted for confounding effects associated with lower grade glioma.
• Higher RT dose to the left cerebrum was associated with poorer verbal memory performance.
• Higher RT dose correlated with hippocampal volume.

## Introduction

Radiotherapy (RT) is an important treatment modality in the management of patients with lower grade glioma (LGG), for whom a long survival can be expected after treatment [1]. Therefore, limiting radiation-induced brain damage (RIBD) is an important goal within the treatment strategy. RIBD can manifest clinically as neuroanatomical changes on imaging and neurocognitive function (NCF) decline [2]. Delaying RT onset and choosing the optimal technique can respectively defer or reduce radiation exposure to the brain and resulting cognitive complications [3]. To date, little is known about RT dose, volume, and timing effect relationships on non-tumour clinical outcomes in LGG patients.

The NCF of patients with LGG is affected by multiple tumour and treatment related factors, hence identifying the RT contribution is complicated. On diagnosis, NCF can already be affected by the tumour and presence of epilepsy [4]. Similar to RT, surgery and medical treatment (chemotherapy and anti-epileptic drugs) can have focal and diffuse effects on patients NCF [5], [6]. In the long term, after tumour progression and multi-modality therapy, the overall NCF function of patients seems most profoundly affected in processing speed, attention and executive function domains [7]. Depending on the tumour location and extension, cognitive domains may either remain intact or show significant deficits. It is important to recognise that a cohort of patients with LGG can, from a functional point of view, be very heterogeneous regarding the variability in tumour location and size [8].

Magnetic Resonance Imaging (MRI) is a valuable tool for detection and quantification of RIBD over time, reflecting pathophysiological mechanisms of radiation response and damage [2], [9]. Standard clinical MRI enables the assessment of several radiological endpoints of RIBD including atrophy and (micro)vascular damage. Atrophy can be quantified on MRI images by measuring the volumes of anatomical brain structures. A number of studies have shown that the loss of hippocampal volume is RT dose-dependent [10], [11], and similar findings are reported for other brain structures [12], [13], [14], [15], [16]. Cerebral microbleeds (CMBs) are a common finding following RT treatment, resulting from (micro)vascular damage [17]. Their incidence and number appear to be time and RT dose dependent as well [18], [19]. It should be noted that changes in brain regions in close proximity to the tumour region are complex to segment and interpret, due to the anatomical changes caused by the tumour and focal treatment.

Advanced RT techniques enable optimisation of treatment plans to obtain higher conformity of the prescribed dose to the target volume and reduced dose to surrounding brain structures. Selective brain sparing can be accomplished using intensity modulated photon techniques. Another emerging RT option is proton therapy, which enables sparing of larger volumes of brain [20], [21], [22]. However, utilisation of technical possibilities to the full extent is hampered by the lack of knowledge on RT dose–effect relationships in the brain and the subsequent clinical relevance [23]. Radiation dose to the hippocampus has been associated with NCF decline [24] and hippocampal sparing RT can result in superior cognitive outcome in patients with multiple brain metastases [25]. It is therefore recommended to limit the RT dose in the hippocampal region [26]. To date, there is insufficient knowledge regarding other pivotal brain regions that should be spared for optimal NCF outcome. The advantageous dosimetry of proton therapy over photons has not yet been translated to a clinical benefit in adult patients with LGG. In order to justify the higher expenses and logistical challenges related to proton therapy, further evidence regarding the impact on clinical outcome is urgently needed.

From a radiation oncology perspective, the clinical relevance of the RT dose bath in the brain is particularly interesting, because advanced RT techniques provide the potential to modify or circumvent dose in this area. We hypothesize that in adult patients with LGG this dose bath contributes to clinically relevant RIBD and is therefore meaningful for patient outcome. In this pilot study, we applied a novel assessment approach to overcome the challenge of identifying brain damage that is RT-induced and avoidable.

We performed a cross-sectional study investigating potential associations between RIBD and RT dose bath in a cohort of patients treated for LGG with photon-RT, using both anatomical radiological features and NCF testing. We divided the patient cohort in more homogeneous subgroups based on tumour location, evaluated RT dose and MRI based endpoints in brain structures contralateral to the tumour, and included the RT clinical target volume (CTV) as a surrogate for focal tumour and treatment effects.

### Material and methods

#### Patients

Medical records were reviewed for eligibility of patients who received RT for LGG between 2007 and 2017 at the University Medical Center Groningen (UMCG), the Netherlands. Patients with mutated isocitrate dehydrogenase (IDH) astrocytoma World Health Organisation (WHO) grade 2 or oligodendroglioma WHO grade 2 and 3 were considered to have LGG. Patients were eligible if they were older than 18 years at diagnosis, at least one year after RT completion and without tumour progression. Enrolled patients provided written informed consent and were scheduled to receive a neuropsychological assessment and MRI scan at a single, cross-sectional time point. This study was approved by the ethics review board of the UMCG (reference number 2018/504).

Thirty-three patients were informed about the study, of whom 19 gave consent. Two patients were excluded, because the study visit was not logistically feasible. Ultimately, 17 patients were available for analysis.

We included non-irradiated healthy controls to facilitate the interpretation of the NCF test performance of patients. Healthy controls were selected and included through professional circuits and matched with the study cohort on age and educational level. The control subjects underwent a neuropsychological assessment under similar testing conditions as the patients from the study cohort, but no MRI scan.

#### Radiotherapy

The clinical radiotherapy treatment planning data with co-registered MRI images were retrieved from medical records. The CTV included the resection cavity and tumour visible on fluid-attenuated inversion recovery (FLAIR) MRI, plus a 10–15 mm margin adapted to anatomical barriers. All patients were treated with photon-RT using 3D-conformal RT (n = 1) or volumetric modulated arc RT/intensity modulated RT (n = 15) or fractionated stereotactic RT (n = 1), using a technique associated PTV margin of 5, 3 and 1 mm respectively. The prescribed dose to the PTV was 50.4–59.4/1.8 Gy. The hippocampus was used for plan optimisation without the use of a specific constraint (ALARA) and was given a lower priority than target coverage.

**Fig. 1:**
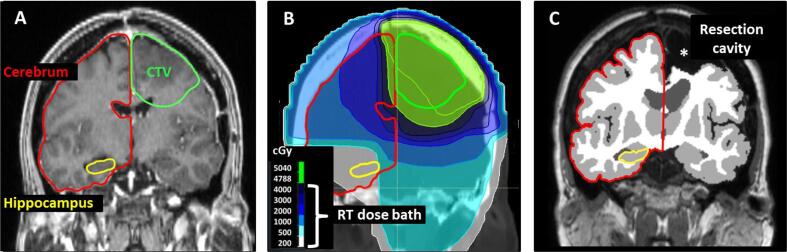
Graphical illustration of the data analysis approach focused on radiological markers of radiotherapy-induced brain damage (RIBD). Markers assessed in the structures exposed to the RT dose bath were structure volume and cerebral microbleeds (CMB)*.* The RT planning MRI **(A)** was co-registered to the RT planning CT with the clinically used photon-RT dose distribution **(B)** and was used to manually delineate structures exposed to the RT dose bath. Outlined structures were cerebrum (red) and hippocampus (yellow) contralateral to the CTV (green), as well as cerebellum (not shown). The volumes from automated segmentation **(C)** and the number of CMB were extracted from the follow-up MRI images for the same set of structures, in order to more precisely correlate RT dose with the RIBD markers and minimize the impact of tumour and/or surgery on the observed RIBD markers.

For each patient, the contralateral cerebrum, contralateral hippocampus and cerebellum were manually delineated on the RT planning MRI according to a contouring atlas [27] and the mean dose in these structures was extracted using Ray Station (8.99, Stockholm, Sweden) (Fig. 1A-B). The delineated structures on the RT planning MRI matched closely with the corresponding structures segmented on the study MRI. The RT planning MRI was performed according to a different scanning protocol, and it was not sufficient for volumetric comparison between baseline and follow-up.

#### MRI imaging

Patients were scanned on a 3Tesla (T) Magnetom Prisma (n = 14), or 1.5 T Magnetom Area (n = 2) or AvantoFit 1.5 T scanner (n = 1) (Siemens Healthineers, Erlangen, Germany). The scanning protocol included a 3D T1 MPRAGE with and without gadolinium (parameters: voxel size 0.9–1.0 × 0.9–1.0 × 0.9–1.0 mm, echo time (TE) 2.32–2.67 ms, repetition time (TR) 2200–2300, inversion time (TI) 900 ms, field of view (FoV) 230–250 mm, flip angle 8°), 3D FLAIR (parameters: voxel size 1.0x1.0x1.0 mm, TE 335–391 ms, TR 5000 ms, TI 1800 ms, FoV 250–260 mm, flip angle 120°) and susceptibility-weighted imaging (SWI) (parameters: voxels size 0.7x0.7x2.0 mm voxel, TE 20–40 ms, TR 28–49 ms, FoV 220–230 mm, flip angle 15°).

Volumes of brain structures were obtained by post-processing of the T1-weighted and FLAIR images using cNeuro cMRI 1.9.3 (Combinostics Oy, Tampere, Finland, www.cneuro.com). CMBs were defined according to the published radiological definition [28] and depicted on SWI images by two experienced observers (JK and AvdH) using Horos, v3.3.5 (Nimble Co LLC d/b/a Purview, Annapolis, MD USA, horosproject.org). For the contralateral cerebrum, contralateral hippocampus and cerebellum, the segmentation-based structure volume and number of CMBs contained within that structure were recorded (Fig. 1C).

#### Neuropsychological assessment

The neuropsychological assessment was performed by trained test assistants who were supervised by a certified clinical neuropsychologist experienced in testing neuro-oncological patients. The assessment included measures in multiple cognitive domains: Symbol Digit Modalities Test (SDMT), Trail Making Test Part A, B and B/A (TMTA, TMTB, TMTB/A), Controlled Oral Word Association Test (COWAT), Rey’s Auditory Verbal Learning Test immediate recall and delayed recall (RAVLT-IR, RAVLT-DR), Verbal Category Fluency, Rey Complex Figure Test (CFT) (Table 1). The total time to complete the assessment was 45 min. Additionally, educational level was scored according to a Dutch classification system [29].

**Table 1:** Overview of the neuropsychological assessment.

Cognitive domain	Test	Description of test and score	Interpretation
**Processing speed and attention**	***Symbol Digit Modalities Test (SDMT*)**	Processing speed, count score	higher score reflects better performance
	***Trail Making Test Part A (TMTA)***	Visuo-motor speed, time score	higher score reflects lower preformance
**Executive functions**	***Trail Making Test Part B (TMTB)***	Visuo-motor speed and cognitive flexibility, time score	higher score reflects lower preformance
	***Trail Making Test Part B/A (TMTB/A)***	Cognitive flexibility, ratio score	higher score reflects lower performance
	***Controlled Oral Word Association Test (COWAT*)**	Phonemic fluency, count score	higher score reflects better performance
**Verbal memory**	***Rey's Auditory Verbal Learning Test - Immediate Recall (RAVLT - IR)***	Verbal memory encoding, count score	higher score reflects better performance
	***Rey's Auditory Verbal Learning Test - Delayed Recall (RAVLT - DR)***	Verbal memory delayed recall	higher score reflects better performance
**Verbal categorical fluency**	***Verbal Category Fluency, Animals***	Semantic fluency, count score	higher score reflects better performance
**Visuospatial function**	***Rey Complex Figure Test (CFT)***	Visuo-construction, count/judgement score	higher score reflects better performance

#### Statistical analysis

The study cohort was compared with healthy controls for differences in: age, using an independent samples *t*-test; gender and dexterity, using chi-square tests; and educational level, using a Kruskal Wallis test. Subsequently, a Mann-Whitney *U* test was used to compare the raw scores of the NCF tests. The study cohort was categorized in patients with left-sided and right-sided tumours. The patient subgroups based on tumour lateralisation were compared with each other using similar methods and were additionally compared for differences in RT treatment factors: CTV, prescribed dose and time interval between RT treatment and study visit using a Mann-Witney *U* test.

To investigate the clinical relevance of the RT dose bath in the brain, the relationship between RT dose and NCF and radiological outcome were analysed using Spearman correlation. In order to account for the confounding focal tumour and treatment effects as much as possible, analysis was performed within the patient subgroups based on tumour lateralisation separately and limited to the contralateral brain structures only. The CTV was included in the analysis as a surrogate for focal tumour and treatment effects.

Statistical analysis was performed using IBM SPSS Statistics for Windows, Version 23.0, Released 2015, Armonk, NY, USA. Non-parametric tests were used for analysis, a two-sided p value of < 0.05 was used for significance.

### Results

The characteristics of the study cohort are listed in Table 2. All patients were right-handed. Compared to healthy controls, the NCF performance of the patients was significantly lower in all cognitive domains (Table 3). The patient subgroup with left-sided LGG (n = 10) had lower antiepileptic drug use than the subgroup with right-sided LGG (n = 7): 60% versus 100%, p = 0.056. The subgroups with left-sided and right-sided LGG did not differ regarding other patient or RT characteristics. The subgroup with left-sided tumours performed significantly lower on verbal tasks: RAVLT-IR and verbal categorical fluency.

**Table 2:** Characteristics of the study cohort.

**Characteristics**				
			**mean (SD)**	**number (%)**
**Patient**	**Age (yrs)**		**47.7 (9.3)**	
	**Gender**	***male***		**11 (64.7)**
		***female***		**6 (35.4)**
	**Epilepsy**	***yes, monotherapy***		**11 (64.7)**
		***yes, polytherapy***		**2 (11.8)**
		***no***		**4 (23.5)**
	**Hypertension**			**0**
	**Diabetes**			**2 (11.8)**
	**Heart disease**			**0**
	**Smoking**			**10 (58.8)**
**Tumour**	**Pathological subtype**	***astrocytoma IDH mutated, WHO grade 2***		**8 (47.1)**
		***oligodendroglioma, WHO grade 2***		**6 (35.3)**
		***oligodendroglioma, WHO grade 3***		**3 (17.6)**
	**Laterality**	***left hemisphere***		**10 (58.8)**
		***right hemisphere***		**7 (41.2)**
	**Location**	***frontal lobe***		**6 (35.3)**
		***temporal lobe***		**6 (35.3)**
		***other***		**5 (29.4)**
**Treatment**	**Surgery**	***single***		**11 (64.7)**
		***multiple***		**5 (29.4)**
		***biopsy only***		**1 (5.9)**
	**Chemotherapy**	***TMZ prior to RT***		**1 (5.9)**
		***PCV sequential to RT***		**15 (88.2)**
		***none***		**2 (11.8)**
	**Radiotherapy**	***3D CRT***		**1 (5.9)**
		***IMRT/VMAT***		**15 (88.2)**
		***Fractionated SRT***		**1 (5.9)**
**Time intervals**	**Diagnosis - study (mo)**		**62.5 (30.7)**	
	**RT treatment - study (mo)**		**40.5 (18.1)**	

**Abbreviations:** Isocitrate Dehydrogenase (IDH), World Health Organisation (WHO), Temozolomide (TMZ), Procarbizine Lomustine Vincristine (PCV), 3 Dimensional Conformal RT (3D CRT), Intensity Modulated RT (IMRT), Volumetric Modulated Arc Therapy (VMAT), Stereotactic RT (SRT).

**Table 3:** Neurocognitive Function test performance of the study cohort, controls and subgroups with left-sided and right-sided tumours.

**Characteristics**		**Study cohort**	**Healthy controls**		**Left-sided LGG**	**Right-sided LGG**	
		**n = 17**	**n = 18**		**n = 10**	**n = 7**	
		**mean (SD)**	**mean (SD)**	**p value**	**mean (SD)**	**mean (SD)**	**p value**
**Patient**	***Age***	47.7 (9.31)	52.8 (8.24)	0.093	48.10 (11.43)	47.14 (5.90)	0.842
	***Gender male, n (%)***	11 (64.7)	7 (38.9)	0.127	5 (50)	6 (85.7)	0.129
	***Educational level***	5.35 (1.16)	6.05 (0.80)	0.089	5.30 (1.06)	5.43 (1.40)	0.594
	***Dexterity right, n (%)***	17 (100)	17 (94.4)	0.324	10 (100)	7 (100)	
	***Antiepileptic drugs yes, n (%)***	13 (76.5)			6 (60)	7 (1 0 0)	**0.056**
**RT treatment**	***CTV (cc)***	183.8 (97.5)			151.07 (60.40)	230.59 (124.66)	0.097
	***Prescibed dose (gy)***	53.26 (3.37)			52.38 (2.99)	54.51 (3.71)	0.186
	***Time post RT (mo)***	40.5 (18.1)			43.00 (22.79)	39.86 (9.46)	0.660
**RT mean dose**	***Contralateral cerebrum (gy)***	17.64 (7.68)			14.98 (7.13)	21.45 (7.22)	0.051
	***Contralateral hippocampus (gy)***	15.01 (12.06)			12.75 (10.09)	18.28 (14.63)	0.435
	***Cerebellum (gy)***	32.17 (7.71)			16.22 (8.19)	11.81 (6.69)	0.242
**Processing speed and attention**	***SDMT***	43.35 (12.18)	56.56 (6.14)	**0.001**	40.00 (14.22)	48.14 (6.84)	0.171
	***TMTA***	40.65 (14.08)	29.56 (10.29)	**0.003**	39.50 (14.73)	42.29 (14.07)	0.961
**Executive functions**	***TMTB***	105.53 (54.87)	64.89 (19.44)	**0.024**	110.40 (59.41)	98.57 (51.37)	0.733
	***TMTB/A***	2.52 (0.84)	2.27 (0.67)	0.363	2.71 (0.90)	2.24 (0.72)	0.222
	***COWAT***	24.29 (9.56)	40.06 (10.76)	**<0.001**	22.20 (10.38)	27.29 (8.04)	0.202
**Verbal memory**	***RAVLT - IR***	34.94 (14.15)	47.17 (10.03)	**0.006**	29.60 (13.95)	42.57 (11.27)	**0.028**
	***RAVLT - DR***	6.41 (3.87)	9.50 (2.57)	**0.012**	5.40 (4.12)	7.86 (3.24)	0.153
**Verbal categorical fluency**	***Animals***	17.59 (5.36)	24.22 (3.96)	**0.001**	15.10 (4.36)	21.14 (4.81)	**0.028**
**Visuospatial function**	***CFT***	30.50 (4.69)	33.14 (2.36)	**0.041**	30.95 (3.26)	29.86 (6.47)	0.769

**Abbreviations:** Lower Grade Glioma (LGG), Clinical Target Volume (CTV), Symbol Digit Modalities Test (SDMT), Trail Making Test Part A, B and B/A (TMTA, TMTB and TMTB/A), Controlled Oral Word Association Test (COWAT), Rey’s Auditory Verbal Learning Test Immediate Recall and Delayed Recall (RAVLT-IR and RAVLT-DR), Rey Complex Figure Test (CFT).

**Fig. 2:**
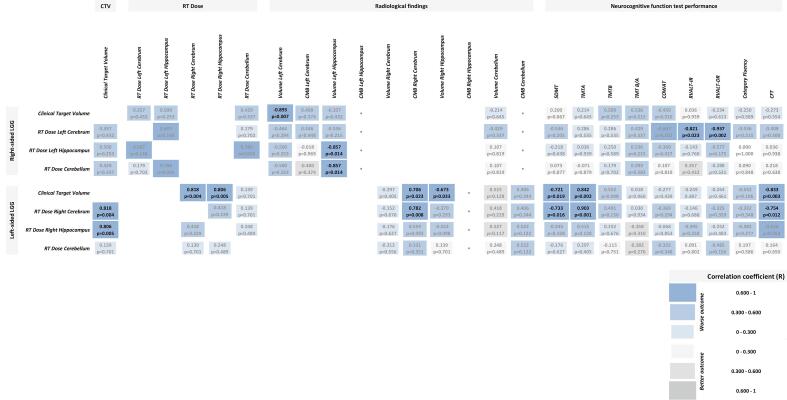
Correlations between RT dose and clinical outcome: radiological findings and NCF test performance, using Spearman correlation test. The results are categorized by patient subgroup based on tumour lateralisation. For each subgroup the correlations between CTV, as a surrogate for focal tumour and treatment effects, and clinical outcome are also shown. RT dose and radiological findings are analysed for cerebrum and hippocampus contralateral to the tumour, and for cerebellum. Darker and larger fields represent higher correlation coefficients (R). Blue fields indicate worse clinical outcome, whereas grey fields indicate better clinical outcome. For all results the R and p values are shown, statistically significant results in bold font. No CMB present.

Correlations of RT dose and CTV with NCF and radiological outcome are shown in Fig. 2. The cognitive test performance scores were not correlated with other patient or tumour characteristics (tumour grade, chemotherapy, cardiovascular risk factors). Use of antiepileptic drugs was significantly correlated with lower performance score on the CFT (r = -0.539, p = 0.025).

#### Left cerebrum and hippocampus – Subgroup with right-sided tumours

**Fig. 3:**
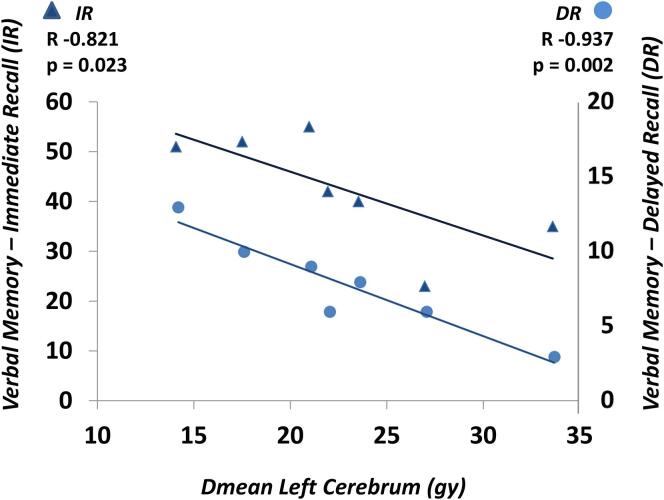
Scatter plot and linear trend line of verbal memory test performance in association with mean RT dose to the left cerebrum. The immediate recall corresponds with the triangles and left y-axis scale, the delayed recall with the dots and right y-axis scale.

Higher RT dose to the left cerebrum was strongly related to poorer verbal memory performance: RAVLT-IR (r = -0.821, p = 0.023) and RAVLT-DR (r = -0.937, p = 0.002), displaying a linear relationship (Fig. 3). Even though RT dose to the left hippocampus did strongly correlate with lower hippocampus volume (r = -0.857, p = 0.014), no correlation was found with hippocampus dose and NCF test performance.

CTV did not have a significant relationship with RT dose to the left-sided brain structures, and no correlations with NCF test performance were found. However, larger CTV strongly correlated with lower volume of the left cerebrum (r = -0.893, p = 0.007).

#### Right cerebrum and hippocampus – Patients with left-sided tumours

Higher RT dose to the right cerebrum related to worse performance on tests for processing speed, attention and visuospatial abilities: SDMT (r = -0.733, p = 0.016), TMTA (r = 0.903, p < 0.001), and CFT (r = -0.754, p = 0.012). Additionally, higher RT dose to the right cerebrum was associated with an increased number of CMB (r = 0.782, p = 0.008). The correlation between RT dose to the hippocampus and hippocampus volume, as seen for the left side, did not reach significance on the right side, and there was no significant correlation with NCF test performance.

Contrary to the subgroup with right-sided tumours, in the subgroup with left-sided tumours, CTV was strongly related with RT dose to the right-sided brain structures: cerebrum (r = 0.818, p = 0.004) and hippocampus (r = 0.806, p = 0.005). Similar to higher right cerebrum dose, larger CTV was significantly correlated to worse performance on tests for processing speed, attention and visuospatial abilities: SDMT (r = -0.721, p = 0.019), TMTA (r = 0.842, p = 0.002), and CFT (r = -0.833, p = 0.003), and higher number of CMB (r = 0.706, p = 0.023). Furthermore, larger CTV correlated with smaller hippocampus volume (r = -0.673, p = 0.033).

#### Cerebellum – Whole cohort

There were no significant correlations between RT dose to the cerebellum and the NCF or radiological findings.

### Discussion

From a radiation oncology perspective, it is relevant and important to obtain further insight into the clinical impact of the RT dose bath outside the target volume, because the distribution of this dose is modifiable using advanced RT techniques. Studying RIBD, clinically manifesting as neuroanatomical changes and NCF decline, in patients with LGG is challenging, since there are multiple factors that can have a profound influence on the outcome, including tumour progression and specifications of multi-modality treatment. The aim of this pilot study was, to investigate RIBD resulting from the RT dose bath more specifically, using both anatomical radiological features and NCF testing. Therefore, we used a novel approach and divided the patient cohort in more homogeneous subgroups based on tumour location, evaluated RT dose and two MRI-based endpoints in brain structures contralateral to the tumour, and included the CTV of RT treatment as a surrogate for focal tumour and treatment effects.

A key methodological step in our study was to create more homogenous patient subgroups based on tumour location. LGG is a diffuse intrinsic brain tumour that can have significant effects on neuroanatomy and NCF. The functional organisation of the brain is complex and can change in response to damage (plasticity of the brain) [30]. Previous studies have shown that patients with LGG can have a different neurocognitive functioning profile based on tumour location and extension, for example verbal functions are mostly affected in patients with left fronto-temporal LGG [8]. Therefore, if a cohort of patients with LGG is not stratified based on tumour location, investigation of radiation effects can be overshadowed by the tumour and focal treatment effects [4]. From our study cohort consisting of right-handed patients only, we were able to show that patients with left-sided tumours performed significantly worse on verbal tasks compared to patients with right-sided LGG. This finding is in line with the reported pre-RT NCF of patients with LGG [31]. The sample size of our study was limited, and categorization of patients in subgroups was only possible based on tumour lateralisation. Future studies with larger sample size should enable a more specific categorization based on tumour lobar location and extension, which could be of interest.

To broaden the evaluation of RIBD, we assessed two radiological endpoints of RIBD in several brain structures, especially those located contralateral to the tumour. For assessment of neuroanatomy, it is interesting to use multiple imaging-based endpoints that reflect different pathophysiological mechanisms of radiation damage response, as these could vary per brain region [2], [9]. In this study we used volumetry and CMB counts as MRI metrics reflecting atrophy and (micro)vascular damage. We observed a potential effect of the RT dose on the number of CMBs, which has previously been described in the literature as increased formation of CMBs with higher RT dose and longer time from RT [18], [19]. This association was found in the subgroup of left-sided tumours only, likely due to the low number of patients in the right-sided subgroup. In contrast with this, we found no CMBs in the hippocampus and instead the changes in hippocampal volumetry seemed to be a more predominant effect of RT dose. These preliminary findings suggest that different radiological metrics (volume, number of CMB) are relevant for different structural brain regions and brain tissue types for the assessment of RIBD. These variations are likely due to a different sensitivity and response of these structures to radiation. For example, RIBD seems to be reflected in some structures predominantly by atrophy and in others by (micro)vascular changes. We feel this is very relevant for both future studies and extrapolation of findings from clinical studies, where we have observed that frequently a single radiological metric is examined (e.g. atrophy, or CMB in isolation), which could lead to incorrect interpretation of results.

In order to elucidate whether the RT dose received by a brain structure is a valid independent risk factor for NCF or radiological outcome, we included CTV in the analysis as a surrogate marker for tumour and the focal treatment effects. In our study, this approach appeared useful for recognising correlations that were potentially confounded. For example, in patients with left-sided tumours, higher RT dose to the right cerebrum was related to worse performance on processing speed and attention tasks. However, in these patients, the CTV also correlated with outcome in these cognitive domains and therefore no definite conclusions on an independent RT dose effect can be made. On the other hand, in patients with right-sided tumours, higher RT dose to the left cerebrum was strongly related to worse verbal memory performance. Interestingly, in line with results from a recently published prospective study [32], we found no relationship between CTV and verbal memory performance, which supports the conclusion that RT dose could be an independent contributor to this outcome.

Hippocampal-sparing RT is already implemented as standard practice in cranial RT. However, superior cognitive outcome with this approach has not yet been demonstrated for patients with LGG. The well-known normal tissue complication probability (NTCP) model for verbal memory outcome based on the dose in 40% of the bilateral hippocampus volume [24], did not perform well in a cohort with LGG [33]. Therefore high-quality NTCP models are not yet available for estimating cognitive endpoints in patients with LGG. In this study we did find a relationship between RT dose to the left hippocampus and hippocampus volume, in line with other studies [10], [11]. However, verbal memory performance was not related to left hippocampus dose, and instead we found a relationship with received dose to the left cerebrum. A possible explanation for this finding could be that a more intricate functional network in the left cerebrum, extending beyond the hippocampus, is important for verbal memory function [34]. The complex relationship between RT dose to the hippocampus and verbal memory function requires further exploration, particularly in patients with LGG, before it can be further implemented for RT technique selection.

Definite conclusions on dose–effect relationships in this study are limited by the small number of patients and cross-sectional design without longitudinal evaluation of changes. To investigate the cerebrum as a whole, without subsequent definition of specific subregions beside the hippocampus, is missing the refinement that is needed for these NCF and MRI evaluations. The lack of high-quality baseline data restrained the investigation of longitudinal RT-induced changes. Furthermore, we cannot rule out bias resulting from multiple testing. Therefore, this work should be regarded as a pilot study that could be important for generating new hypotheses and strategies for future studies.

In order to further investigate RIBD in patients with LGG, it is necessary to conduct prospective cohort studies with standardised NCF assessments and multi-parametric MRI protocols, preferably performed on the same type of MRI scanner, at multiple time points during the disease course. In addition, the multi-parametric radiomics algorithms should be developed and tested for the analysis of brain tumours. As LGG is a low-incidence disease, collaborative efforts are essential to obtain large study cohorts. Over the last years, the neuro-oncology group of the European Particle Therapy Network (EPTN) has initiated the publication of consensus reports to facilitate collaboration and further propel the field [26], [27], [35]. Beside the focus on various brain subregions by using anatomical imaging, it would be very valuable to gain more insight into neuronal networks by using functional imaging techniques [36]. Ultimately, the goal is the development of high-quality NTCP models for RIBD endpoints that can be used to select the most optimal RT treatment plan, for example proton therapy [23] or other technical innovations in the future such as ultra-high dose rate (FLASH) RT [37] for patients with LGG and other brain tumours.

In conclusion, we used a novel approach to detect and quantify RIBD more specifically by combining MRI and NCF outcome endpoints, while accounting for the confounding effects of the tumour and treatment. We observed a potentially independent RT dose effect on the left-sided brain structures, exhibiting as verbal memory performance decline correlating with hippocampal volume. The results of this pilot study support our hypothesis that the low-dose bath, typical for photon-RT, contributes to clinical outcome in patients with LGG. Future studies on RIBD in patients with LGG could benefit from the approach in this study in terms of both design and interpretation of brain structure specific results.

## Funding

This study was financially supported by the UMCG Cancer Research Fund.

## Declaration of Competing Interest

The authors declare that they have no known competing financial interests or personal relationships that could have appeared to influence the work reported in this paper.
